# The fMRI correlates of visuo-spatial abilities: sex differences and gender dysphoria

**DOI:** 10.1007/s11682-022-00638-5

**Published:** 2022-04-06

**Authors:** Gioele Gavazzi, Alessandra Daphne Fisher, Stefano Orsolini, Andrea Bianchi, Alessia Romani, Fiorenza Giganti, Fabio Giovannelli, Jiska Ristori, Francesca Mazzoli, Mario Maggi, Maria Pia Viggiano, Mario Mascalchi

**Affiliations:** 1 IRCCS SDN, Naples, Italy; 2 “Mario Serio” Department of Experimental and Clinical Biomedical Sciences, University of Florence, Florence, Italy; 3 Department of Electrical, Electronic, and Information Engineering “Guglielmo Marconi”, University of Bologna, Cesena, Italy; 4 Neuroradiology Unit, “Careggi” University Hospital, Florence, Italy; 5 Department of Neuroscience, Psychology, Drug Research, Child Health, University of Florence, Via San Salvi, 12, 50135 Florence, Italy

**Keywords:** Gender dysphoria, V1, V5, Visuospatial abilities, Transgender people

## Abstract

The contribution of brain regions to visuospatial abilities according to sex differences and gender identity is inconsistently described. One potential explaining factor may be the different tasks employed requiring a variable load of working memory and other cognitive resources. Here we asked to 20 cis and 20 transgender participants to undergo functional Magnetic Resonance Imaging during performance of a judgement line of orientation test that was adapted to explore the basic visuospatial processing while minimizing the working memory load. We show that V1 activation may be viewed as a brain area with enhanced activation in males, regardless of participants’ gender identity. On its turn, gender identity exclusively influences the visuospatial processing of extrastriate visual areas (V5) in women with gender dysphoria. They showed enhanced V5 activation and an increased functional connectivity between V5 and V1. Overall our neuroimaging results suggest that the basic visuospatial abilities are associated with different activations pattern of cortical visual areas depending on the sex assigned at birth and gender identity.

**Supplementary Information:**

The online version contains supplementary material available at 10.1007/s11682-022-00638-5.

Over the past 30 years innovative technologies and methods have generated a growing pile of studies exploring gender differences in brain functioning and anatomy (Eliot et al., 2021). Among the explored cognitive domains, the visuospatial abilities hold a prominent position. In particular, several studies have shown sex-based differences in the visuospatial domain with men performing better than women (Vandenberg & Kuse, 1978; Linn & Peterson, 1985; Johnson & Meade, 1987; Goldstein et al., 1990; Voyer et al., 1995; Karádi et al., 2003; Peters, 2005). Neuroimaging investigations revealed that during a Mental Rotation Test (MRT), men activate parietal cortex more than women who, in their turn, exhibit a more marked activation in the inferior frontal cortex (Hugdahl et al., 2006). Such activation patterns might depend on divergent strategies adopted to accomplish the task (Burke et al., 2016; Hugdahl et al., 2006). Namely, men might be inclined to use more holistic strategies, while women might prefer verbal or analytical strategies. Most of the purposely designed studies have shown sex-based differences, but the neural correlates of these differences have not been discovered yet.

It has been suggested that performance-related factors independent of sex might account for the failure to show sex differences of several studies that used other tests than MRT (Unterrainer et al., 2000). Although environmental, biological and neurobiological factors have been considered among the possible causes of sex-related differences in behavior and cortical activations (Hugdahl et al., 2006; Kimura, 1996), the level of task complexity, namely complex vs. basic perceptual tasks, might also play a decisive role. In fact, variants of MRT and other complex tasks including map reading tests (Beatty, 2002) and road indication tests (see Voyer et al., 1995 for meta-analysis) are the most commonly used tasks capable of revealing sex differences. On the other hand, inconsistent results were obtained using the Judgment Line of Orientation test (JLO) whose complex or basic design depends on the version of the task itself (Benton et al., 1975; Saykin et al., 1995; Clements et al., 2006; Clements-Stephen et al. 2009; Lippa et al., 2010). In particular, the classic behavioral version of JLO (Benton et al., 1975), similarly to the MRT, is a complex task involving visuospatial processing and remarkable working memory (WM) load. Hence, using this kind of task to explore differences in visuospatial processing could be confounded by the impact of task load.

The evaluation of sex differences in visuospatial processing has been extended, in recent years, to individuals with Gender Incongruence (GI), defined as the discrepancy between the experienced gender and the sex assigned at birth (WHO). The clinically significant distress—which may result from this incongruence—is usually defined as Gender Dysphoria (GD). Notably, the idea of the sexual dimorphic brain as the anatomical substrate of psychosexual development has been widely explored and research has focused on the influence and shaping role of genes and gonadal hormones on sexual morphological or funtional differentiation of the brain (Bao & Swaab, 2011; Cohen-Bendahan et al., 2005; Kreukels & Guillamon, 2016; Ristori et al., 2020). However, a recent review propose that the sexual dimorphic brain does not exist, suggesting that the reported behavioral differences between men and women cannot be ascribed to anatomical differences once the individual brain size is taken into account for (Eliot et al., 2021). However, apart from the anatomical level, it is conceivable that brain sexual functional dimorphism, could also be observed in cognitive processes that show sex difference as visuospatial abilities. In this regard, a controlled study of cis gender men and women vs. people with GD/GI may be a valuable experimental setting to better understand the different visuospatial abilities between the male and female brain, and to explore the possible role of gene, gonadal hormones and cultural aspects on gender identity.

Along this line, Burke et al. (2016) investigated visuospatial abilities with functional MRI (fMRI) during execution of the MRT in girls with GD, control girls and boys. They observed that the brain activation patterns of hormone-naive girls with GD were different from those observed in control girls and resembled those in control boys, suggesting a masculinization of functions associated with visuospatial working memory in girls with GD. To explain these results, the authors speculated that GD females and boys share similar interests and preferences for certain hobbies and activities reflecting different experiences and training of visuospatial abilities (Burke et al., 2016).

In the present prospective study, we explored with fMRI two groups of control samples (10 cisgender women and 10 cisgender men) and of people with GD (10 transgender women and 10 transgender men) during execution of an automatic version of JLO task that has been introduced with the purpose of minimizing involvement of additional resources as WM (Kesler, 2004; Clements et al., 2006; Clements-Stephen et al., 2009).

The aim of the present study is to investigate i) whether this specific version of the JLO can capture differences in brain activations between cisgender subjects; ii) whether men and women with GD show modified activation patterns.

## Methods

### Participants

The study was approved by the local ethics committee (2013/0016117) and written informed consent was obtained from each participant in conformity with the Helsinki Declaration and later amendments or comparable ethical standard.

Forty subjects, namely 20 cisgender individuals (10 cismen, 10 ciswomen; hereafter CM and CW respectively) and 20 trans individuals (10 transmen, 10 transwomen, hereafter respectively TM and TW) of similar age (CM mean ± SD age = 27.1 ± 1.62 years; CW mean ± SD age = 27.6 ± 3.24 years; TM mean ± SD age = 32.8 ± 12.96 years; TF mean ± SD age = 29.3 ± 7.60 years) and education (CM mean ± SD age = 27.1 ± 1.62 years; CW mean ± SD age = 27.6 ± 3.24 years; TM mean ± SD age = 32.8 ± 12.96 years; TF mean ± SD age = 29.3 ± 7.60 years) were examined (see Supplemetary Table 1 for further details).

The 20 trans individuals belonged to a consecutive series referring for the first time to the gender clinic at Florence University Hospital. They were enrolled in the present study if they met the following inclusion criteria:

- Age older than 18 years.
- Diagnosis of GI/GD based on formal psychiatric classification criteria and performed through several sessions with two different mental health professionals specialized in GI/GD.

The exclusion criteria were:

- Genital affirming surgery performed
- Illiteracy
- Mental retardation
- History of psychiatric or neurological diseases

The 20 subjects of the cisgender groups were enrolled by means of local advertisement at the University of Florence and met the following inclusion criteria: age older than 18 years, absence of a GI/GD or psychiatric disorders. The exclusion criteria were:

- The use in the previous 6 months of any hormonal treatment
- Illiteracy
- Mental retardation
- DSD
- History of psychiatric or neurological diseases
- Pregnancy or current lactation

All participants were blind to the purpose of the study, right-handed and had normal or corrected-to-normal vision. Participants were screened to ensure that they satisfied MRI safety requirements and showed no structural brain abnormalities on MRI sequences obtained before the fMRI task.

### Raven matrices assessment

Since Raven matrices correlate with visuo-spatial abilities (Johnson, 1990), prior to fMRI experiment, two psychologists evaluated all participants using the Raven Standard Progressive Matrices (John and Raven, 2003).

### Experimental paradigm

Participants viewed stimuli on a MRI-compatible display system (SensaVue fMRI, Invivo Corporation, Gainesville, FL,USA) by means of a mirror attached to the head coil. The task was an adapted version (Clements et al., 2006; Kesler, 2004) of the JLO that minimizes the contribution of working memory. The entire experiment was preceded by a training session composed by a block of 12 trials to ensure understanding of the instructions. The protocol was composed by Rest, Experimental (E) and Control (C) epochs of the task, according to this sequence: Rest – E – C – E—C – E – C – Rest. Briefly, there were two Rests, three Experimental and three Control epochs. Each Rest epoch lasted 30 s during which participants fixed a cross located in the middle of the screen. Control epochs began with a 4 s display of the instruction “Judge if colour matches”. Similarly, Experimental epochs began with the instruction “Judge if orientation matches”. All conditions were equally balanced among trials.

**Fig. 1:**
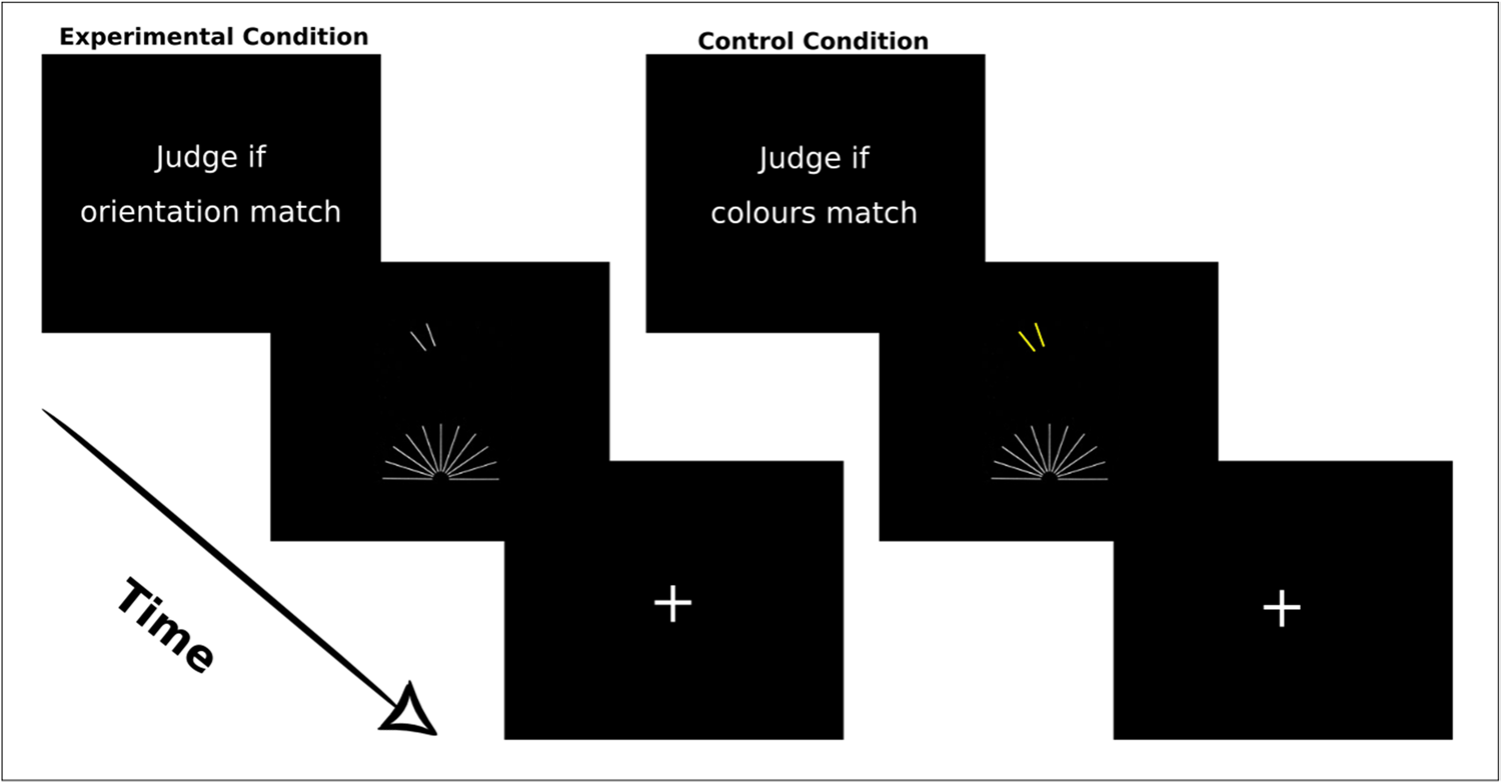
Stimuli Protocol. Each Control and Experimental condition began with a 4 s display of the instruction (“Judge if colour matches” or “Judge if orientation matches”, respectively). It followed a fan of 11 lines displayed (9 white and 2 yellow) at the bottom of the screen and two yellow line displayed above for 1 s. In the Experimental condition, these two yellow lines could be displayed in either the same or different orientation as the two yellow lines in the fan. In the Control Condition subjects were requested to identify if the lines above the fan, in this case either displayed as white or yellow, were the same colour as the fan of 11 lines. Depending on the condition, participants had to press the button with their right index finger whether the colours or the orientation between the fan and the two lines displayed above corresponded

The visual stimulation consisted of a fan of 11 white lines displayed at the bottom of the screen (Fig. 1) and two yellow lines displayed above. In the Experimental condition, these two yellow lines could be displayed in either the same or different orientation as the two yellow lines in the fan. Participants had to press a button with their right index finger if the top and bottom yellow lines orientation corresponded. The Control condition consisted of a colour discrimination task, controlling for basic visual discrimination abilities and participants compliance to the protocol. Subjects were requested to identify if the lines above the fan, either displayed as white or yellow, were the same colour as the fan of 11 lines (all white). Participants pressed the button with their right index finger whether the colours between the fan and the two lines displayed above corresponded.

### MRI data acquisitions

MRI acquisitions were performed on a 3 T scanner (Ingenia, Philips Healthcare, The Netherlands) equipped with Omega HP gradients with maximum amplitude of 45 mT/m and slew rate of 200 T/m/s for each axis and a 32-phased-array-element head coil. After scout and T2 weighted FLAIR images to check for possible brain abnormalities, 3D T1-weighted imaging and fMRI were obtained in all subjects.

T1-weighted MR images were acquired with a sagittal high-resolution 3D sequence (repetition time [TR]/echo time [TE]/inversion time [TI] = 8/3.7/925.6 ms, flip angle [FA] = 8°, slice thickness = 1 mm, field of view [FOV] = 240 mm × 240 mm, number of slices = 149, matrix size = 240 × 240).

For the fMRI experiment we utilized a T2*-weighted echo-planar imaging (EPI) sequence with parameter setted similarly to previous studies from our group (Gavazzi et al., 2017, 2019—TR/TE = 3000/35 ms, FA = 90°, slice thickness = 3.5 mm, FOV = 240 mm × 240 mm, number of slices = 42, matrix size = 240 × 240). One hundred and one scans were acquired, for a total acquisition time of about 5 min, from which the first 5 scans were discarded.

### MRI data analysis

Volumes acquired with fMRI were analyzed using the FMRIB Software Library (www.fmrib.ox.ac.uk/fsl). Canonical preprocessing was applied (first 5 time points removed, slice-time correction with custom timings, motion correction, and intensity normalization). We adopted the following filtering steps: temporal high-pass with cutoff at 50 s; spatial smoothing using a 2 mm full width half maximum Gaussian kernel. Co-registration of fMRI images to the individual high-resolution T1-weighted image was performed using a 6-degree of freedom registration. The individual high resolution T1-weighted images were co-registered to the standard space Montreal Neurological Institute 152 (MNI152) brain with an affine transformation (12 degree of freedom) followed by a nonlinear transformation. fMRI images were co-registered to the MNI152 standard space using the transformation previously computed when co-registering the individual high-resolution T1-weighted images to the MNI152 standard space 1 mm T1-weighted template.

Time points in the fMRI data set that were affected by large motion, namely displacement > 1.5 mm of the absolute mean displacement, were identified from motion correction parameters (motion correction FMRIB's Linear Image Registration Tool) and accounted for in a confound matrix at the subject-level analysis. Each stimulus delta functions sequence was convolved with a double gamma hemodynamic response function, whereas the temporal derivative was included in the model and temporal filtering applied.

To explore activity related to the execution of the JLO task, a General Linear Model (GLM) of ORIENTATION vs COLOUR contrast was set at subject-level analysis including a distinct explanatory variable to account for baseline volumes. Each model explanatory variable was convolved with a double gamma hemodynamic response function, whereas temporal derivatives were included, and temporal filtering applied.

Because the experimental design involved randomized intervals stimuli, we reduced autocorrelation in the data by applying voxel-wise pre-whitening. To establish between-group differences, we used an unpaired t test with a mixed effects model where age was included as nuisance covariate. All group analyses were performed in the MNI152 standard space. For all statistical analyses the resulting Z (Gaussianized T/F) statistic images were thresholded using clusters determined by Z > 2.3, and a corrected p-value < 0.01. To anatomically map the significant clusters in the resulting Z statistic images probabilistic atlases were used (Desikan et al., 2006; Eickhoff et al., 2007; Diedrichsen et al., 2011).

To explore possibly abnormal patterns of activation, we also performed a functional connectivity (FC) analysis of the areas exhibiting significantly different BOLD contrast in the GI/GD participants when compared with their respective cisgender groups (TW vs CM and TMvs CW). To this aim we quantified relevant coupling of a cortical ROI to the rest of the brain in a voxelwise analysis using a Task-residual functional connectivity approach (Fair et al., 2007).The ROI was selected from the Jüelich histological atlas (Eickhoff et al., 2007) and for each subject the fMRI sequence residual of the previous GLM analysis was transformed to the MNI152 standard space. From such a residual sequence, containing the BOLD signal after that task related effects were regressed out, the average time course of the selected ROI was extracted and used to calculate the linear correlation with every voxel within the brain mask. Correlation values contained in the resulting volumes represent the background connectivity associated to the experimental task (Elkhetali et al., 2019).

To establish group differences in values of the background connectivity, we investigated significant between-group positive and negative statistics using unpaired t-test and permutation-based non parametric inference within the GLM framework where age was included as nuisance covariate.

For each GLM contrast, p-values were calculated employing permutation-based statistics (1,000 permutations) and corrected for multiple comparisons with threshold-free cluster enhancement (TFCE) method (Smith & Nichols, 2009). A p-value < 0.01 corrected for multiple comparisons across space (family-wise error rate correction) was considered statistically significant.

## Results

### Age, education and raven matrices assessment

One-way ANOVA [F(3,36) = 1.09, *p* = 0.37] showed no statistically significant difference of age among the four groups.

Another one-way ANOVA [F(3,36) = 10.93, *p* < 0.01] showed a statistically significant difference of education among groups. Multiple post-hoc pair-wise comparisons corrected with Bonferroni method revealed that CM and CW showed statistically significant higher scores than TM and TW (respectively—*p* = 0.004 and *p* < 0.001).

For this reason Raven matrices assessement’ score was corrected with education and therefore computed on a percentile basis in 1–5 grades. A one-way ANOVA [F(3,36) = 5.005, *p* = 0.005] showed a statistically significant difference of scores (corrected for education) among the four groups of subjects. Multiple post-hoc pair-wise comparisons corrected with Bonferroni method revealed that only CM showed statistically significant higher scores than TW (*p* = 0.004). See Supplemetary Table 1 for further details.

### Task performance

Behavioural data are summarized in Table 1. There were no statistically significant differences among groups as determined by one-way ANOVA—F(3,36) = 0.6250, *p* = 0.60.

**Table 1:** Behavioural performances. The groups differences in behavioral accuracy for Orientation and Color discrimination were not statistically significant by one-way ANOVA

	Orientation		Color	
Group	Accuracy	SD	Accuracy	SD
CM	0.57	0.09	0.98	0.02
CW	0.5	0.12	0.99	0.01
TM	0.47	0.11	0.99	0.01
TW	0.49	0.20	1.00	0.00

### Brain activations

**Fig. 2:**
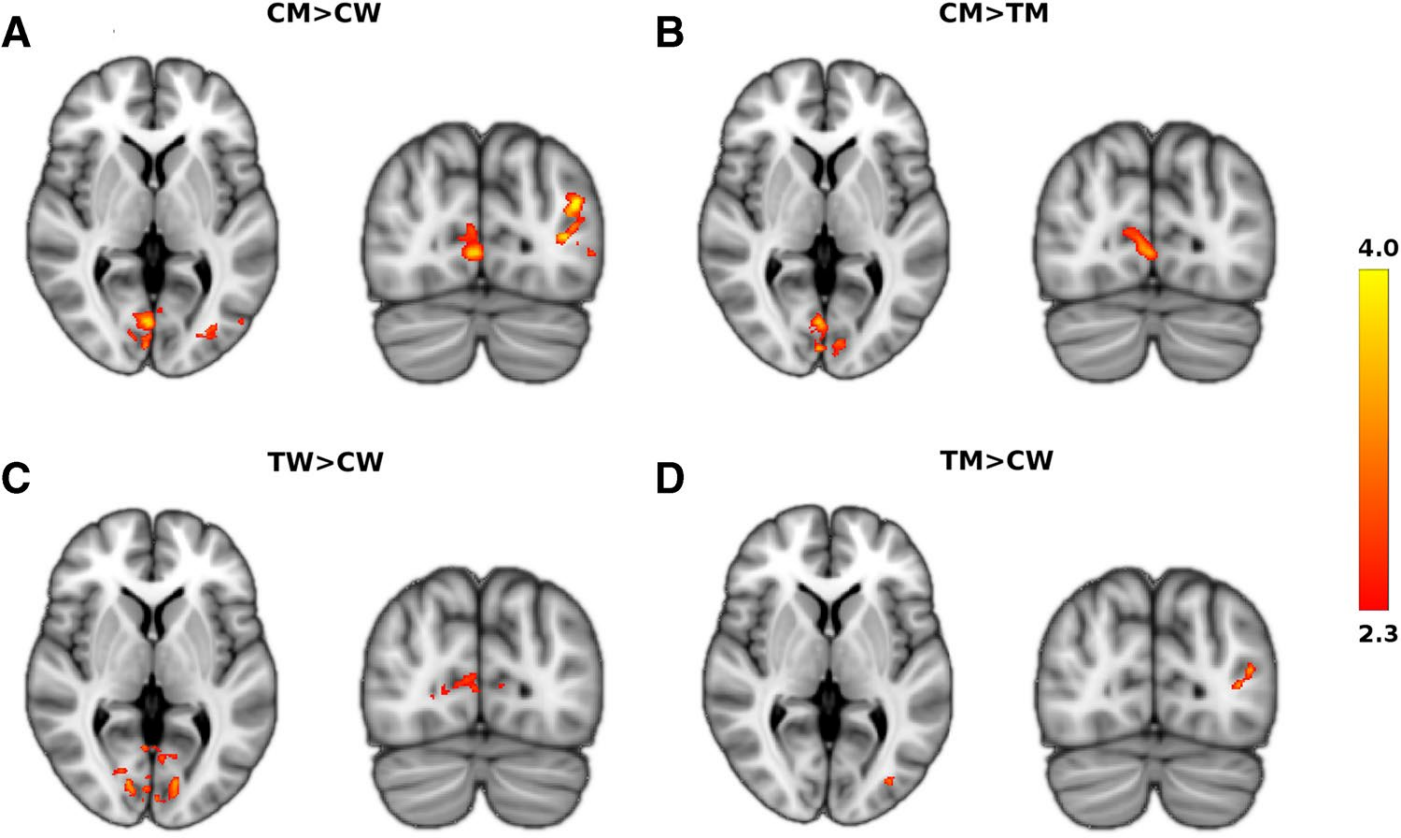
Between Groups Analysis. Clusters of brain activation show between groups differences in the Primary Visual Cortex (V1) for cisgender men contrasted with cisgender women (CM > CW), cisgender men contrasted with transgender man (CM > TM) and transgender women contrasted with cisgender women (TW > CW) and in the Inferior Parietal Lobule (IPL) for cisgender men contrasted with cisgender women (CM > CW) and cisgender men contrasted with transgender women (CM > TW). Visual Cortex V (V5) is found for transgender men and cisgender women (TM > CW) contrast. These results are detailed in Table 2

Clusters of significantly higher activation when comparing the four groups of subjects are detailed in Table 2 and shown in Fig. 2. The CM > CW contrast yielded two clusters, the Primary Visual Cortex (V1) and left Inferior Parietal Lobule (IPL). The CM > TM contrast revealed the left IPL and the right V1. The TW > CW contrast showed the right V1. Finally, TM > CW contrast demonstrated left Visual Cortex V (V5). No significant differences were observed in TW vs CM contrast.

**Table 2:** Between Groups Analysis. Cluster of significant (p-value < 0.01) between groups differences in brain activation for the listed contrasts. Analysis cluster formation threshold was set at 2.3 of z-stat value. Coordinates are reported in MNI space

Group	Region	z-MAX	z-MAX X (mm)	z-MAX Y (mm)	z-MAX Z (mm)	Voxels (mm^3^)
CM > CW	Visual cortex V1 R	3.96	3	-77	2	3475
	Inferior parietal lobule L-IPL	4.55	-38	-75	9	1366
CM > TM	Inferior parietal lobule L-IPL	4.13	-59	-51	28	2951
	Visual cortex V1 R	3.85	16	-80	7	2334
TM > CW	Visual cortex V5 L	4	-35	-81	11	944
TW > CW	Visual cortex V1 R	4.3	1	-84	11	5257

No clusters of significantly decreased activation were found in all comparisons.

**Fig. 3:**
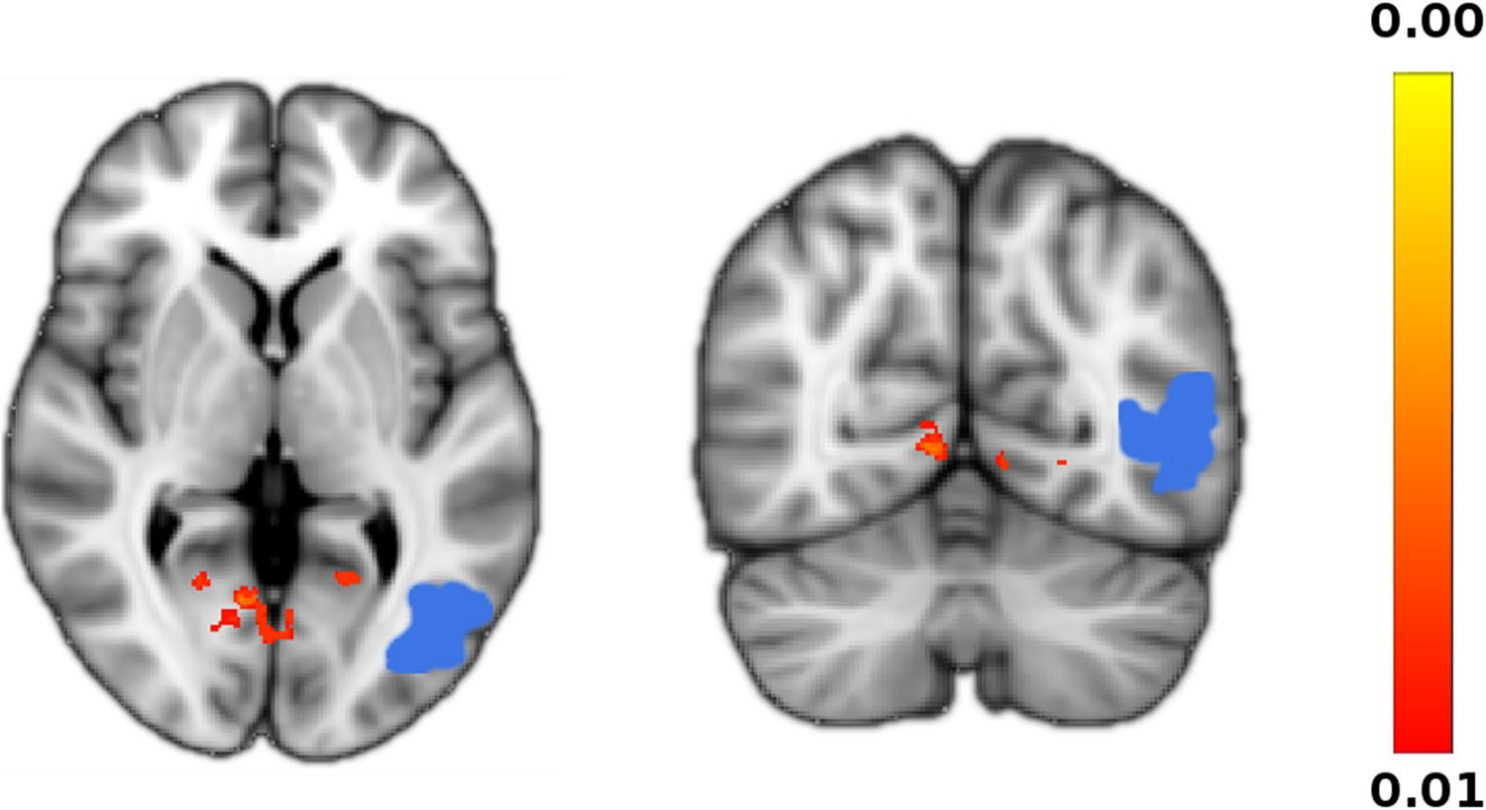
Functional Connectivity from l-V5 for contrast TM  >  CW. The functional connectivity analysis reveals increased connectivity of left V5 (indicated in blue) with bilateral V1 and Superior Parietal Lobule (SPL) -not shown here- for the TM group against the CW group. More details are reported in Table 3

The FC analysis of the left Visual Cortex V (V5) revealed by TM > CW contrast demonstrated increased connectivity between the same area with bilateral V1 and Superior Parietal Lobule (SPL) – see Table 3 and Fig. 3.

**Table 3:** Functional Connectivity from l-V5 for contrast TM  >  CW. The functional connectivity analysis revealed increased connectivity for the TM group against the CW group between left V5 and 8 clusters contained in the structures listed. Functional Connectivity maximum value within each cluster is reported with MNI coordinates and cluster size

Region	MAX X (mm)	MAX Y (mm)	MAX Z (mm)	Voxels (mm^3^)
Visual cortex V1 BA17 L/R	8	-64	1	610
Visual cortex V1 BA17 L	-17	-57	-3	353
Visual cortex V1 BA17 R	18	-61	8	155
Visual cortex V1 BA17 R	17	-59	-1	110
Superior parietal lobule 7A R	6	-58	50	105
Superior parietal lobule 7A L	-9	-56	53	68
Visual cortex V1 BA17 L	-8	-66	-3	57

## Discussion

Research focusing on visual-spatial abilities as a function of sex and gender identity has been conducted mainly using *spurious* tasks, such as MRT, which require significant involvement of WM (Burke et al., 2016). Herein we explored the visuo-spatial abilites of cisgender and transgender subjects using an adapted version of the JLO task in which the contribution of WM is minimized. Consistent with previous studies (Clements et al., 2006; Clements-Stephens et al., 2009), the behavioral data did not differ between the two groups of cisgender and transgender in the adapted JLO task. However, notably fMRI results revealed partially different patterns of brain activation in the four groups that were matched for age and performances. In fact, the left inferior parietal lobule was observed differently activated exclusively for cismen when compared to ciswomen (CM > CW and CM > TM), but not in transwomen (assigned males at birth) when compared to ciswomen (TW > CW). It is remarkable that the activation of parietal lobe was in the left hemisphere instead of the right one, differently from what expected according to literature on visual spatial abilities (Franzen, 2000). The fact that our participants’ performance was not statistically different and that the inferior parietal lobe has been reported as a potential marker of the transgender condition, both in morphometrical studies (Mueller et al., 2021) and in fMRI studies with different tasks (e.g. Burke et al., 2016; Fisher et al., 2020), may suggest that this region is not specifically triggered by the pure visuo-spatial abilities. For the above reasons, one might hypothesize that the left inferior parietal lobule can mediate a mechanism not recruited exclusively by visuo-spatial abilities but in several cognitive processes, representing probably a putative marker of the (cis) male gender rather than a marker of the GI/GD condition.

At variance, regardless of the perceived gender identity, when assigned male at birth subjects (both CM and TW) were compared to cis, the primary visual cortex V1 was more activated (Table 2 and Fig. 2). The finding that in assigned male at birth subjects, V1 is differently activated by contrasting two different visual activities with the same visual content is an intriguing result. It suggests that differences in visuospatial elaborations between assigned male at birth individuals and the other groups do not involve solely higher cortical areas (parietal lobe), but can be detected since the very first level of the visual hierarchy of spatial processing.

When contrasting the transgender groups with the corresponding cisgender groups (of the same sex assigned at birth: TW vs CM and TM vs CW) we observed a statistically significant higher activation of V5 in the TM > CW contrast. Notably, this difference was not observed in the mirror contrast between the groups with male sex assigned at birth (i.e. TW vs CM).

Overall, considering the above results concerning the sex assigned at birth and those related to the perceived gender identity, one might hypothesize the existence of a link between the higher V1 activation observed in subjects with male sex assigned at birth (both CM and TW) and the increased activation of V5 in females assigned at birth with perceived male gender identity (TM). In fact, taking into account both the absence of differences in behavioral performance between the groups and the enhanced functional connectivity between V5 and V1 (Table 3 and Fig. 3) in TM, one may speculate that these participants could hyper-activate V5 to feed the activation of V1 observed in all male at birth groups (CM and TW).

This hypothesis is supported by the V5 activation that has been observed also in other tasks that stress both visuospatial abilities and WM, suggesting that V5 activation does not seem involved in the visuospatial processing *per se,* but rather in the WM elaboration of information during complex visuospatial processing (Podzbenko et al., 2002; Campana et al., 2005). In particular, since we used a JLO version that minimizes WM load and we observed no difference in behavioral performances among the four subject groups, in our opinion the V5 activation in TM participants is unlikely to reflect the basic visuospatial abilities or the WM required by the task. Rather, we submit that V5 higher activation in TM subjects may be an epiphenomenon associated with the general attempt of these subjects to enhance their visuospatial abilities usually implying a remarkable WM load (see below), even if the WM load in the particular JLO task we employed is minimized.

How can our results be integrated within the debate concerning genetic and cultural factors underling GI/GD? We observed that CM show a higher activation of V1 than CW, regardless the perceived gender identity. At variance, GI/GD seems to be associated in TM with a hyper-activation of V5 as well as with an enhanced functional connectivity between this area and V1 (TM > CW). Hence, V1 functioning in visuospatial processes might resemble that of other typical sexual dimorphic brain areas (Hill et al. 2005). Indeed, a sexual dimorphic visuospatial functioning seems reasonable because V1 is well-known for being an area devoted to the elaboration of the orientation of visual stimuli (Adesnik et al., 2012; Iacaruso et al., 2017; Park et al., 2019; Sun et al., 2016) and because it was reported to have a different connectivity in males and females (Adesnik et al. 2010; Ritchie et al., 2018). Interestingly, a substantial consistency of WM networks in males and females was reported in a recent meta-analysis with minor dimorphic modulation in the same and additional areas, including prefrontal and limbic regions, right superior parietal lobe, the left insula, bilateral thalamus and the cerebellum (Hill et al., 2014). However, none of these areas were revealed by our analysis and this is in line with the idea that the differences we observed should not be ascribed to WM, but, mostly, to the visual stimuli processing in V1.

Differently, in our study the GI/GD condition was exclusively associated with a hyperactivation of V5 in TM. This result might reflect contribution of non-genetic factors. In fact, if a genetic background would have a prominent role, we should have observed the same increased activation comparing transgender women with cisgender men. Actually, considering the role played by V5 in WM, we may speculate that its hyperactivation in transgender men (when compared with the cisgender women) represents an epiphenomenon related to the general effort of these individuals to resemble the male brain functioning. This possibility is corroborated by the fact that the SPL was the only brain region, beside V1, that we found to be enhanced in our FC analysis of V5 and this region is well-known also to absolve WM related functions (Koenigs et al., 2009). Factors triggering V5 activation are still unknown. However, present results suggest that V5 activation may not be entirely related to genetic factors. The influence on gender role exerted by social clues—for instance the belief that men are better than women in visuospatial tasks and STEM classes, (Reich et al., 2018)—is a reasonable hypothesis to be explored in future studies. In particular, it would be interesting to explore investigate the hyperactivation of V5 in TM, taking into account that these transgender participants tend to play more male games than CW during the development of brain connectivity. Ultimately, the activities performed during the development of brain connectivity (sport, video-games and spatial toys preferences) could shape behavioural visuospatial abilities and the related brain activation (Voyer et al., 1995). Intriguingly, it has been recently shown that sex differences in visuospatial tasks can be effaced by taking into account spatial toys and sport expositions during childhood—with males at birth performing these activities more frequently than females (Doyle et al., 2012; Gold et al., 2018).

We recognize as a limitation of the present study is the relatively small-size of the sample analyzed from a single center study. In addition, our results may be flawed by the lower score at the Raven matrices of TW subjects as compared to CM. However this did not translate into significant differences in the behavioral performance and V1 activation. Moreover, the Raven matrices score of TM and CW subjects, the former showing enhanced V5 activation when compared to the latter, was similar.

In conclusion, our fMRI study reveals that in absence of behavioral differences in an adapted JLO that minimizes WM load, assigned male at birth subjects show a hyperactivation of V1, when compared to assigned female at birth subjects. V1 might hence be added to other genetically-determined areas of the brain with different functioning according to sex/gender. The V5 activation observed in women with GI/GD might represent an epiphenomenon of the enhanced visuospatial abilities requiring relevant WM load, possibly related to an hypothetical childhood preference of TM for male gender roles (including games/activity).

## Supplementary Information

Below is the link to the electronic supplementary material.

Supplementary file1 (XLS 22 kb)

## Data Availability

The data that support the findings of this study are available from the corresponding author upon request.
